# Histone deacetylase inhibitors promote glioma cell death by G2 checkpoint abrogation leading to mitotic catastrophe

**DOI:** 10.1038/cddis.2014.412

**Published:** 2014-10-02

**Authors:** M Cornago, C Garcia-Alberich, N Blasco-Angulo, N Vall-llaura, M Nager, J Herreros, J X Comella, D Sanchis, M Llovera

**Affiliations:** 1 Cell Signaling and Apoptosis Group, Institut de Recerca Biomèdica de Lleida (IRBLleida), Universitat de Lleida, Lleida, Spain; 2 Calcium Signaling and Neuronal Differentiation Group, IRBLleida, Universitat de Lleida, Lleida, Spain; 3 Institut de Recerca de l'Hospital Universitari de la Vall d'Hebron (VHIR), Institut de Neurociències, Universitat Autònoma de Barcelona and Centro de Investigación Biomédica en Red sobre Enfermedades Neurodegenerativas (CIBERNED), Barcelona, Spain

## Abstract

Glioblastoma multiforme is resistant to conventional anti-tumoral treatments due to its infiltrative nature and capability of relapse; therefore, research efforts focus on characterizing gliomagenesis and identifying molecular targets useful on therapy. New therapeutic strategies are being tested in patients, such as Histone deacetylase inhibitors (HDACi) either alone or in combination with other therapies. Here two HDACi included in clinical trials have been tested, suberanilohydroxamic acid (SAHA) and valproic acid (VPA), to characterize their effects on glioma cell growth *in vitro* and to determine the molecular changes that promote cancer cell death. We found that both HDACi reduce glioma cell viability, proliferation and clonogenicity. They have multiple effects, such as inducing the production of reactive oxygen species (ROS) and activating the mitochondrial apoptotic pathway, nevertheless cell death is not prevented by the pan-caspase inhibitor Q-VD-OPh. Importantly, we found that HDACi alter cell cycle progression by decreasing the expression of G2 checkpoint kinases Wee1 and checkpoint kinase 1 (Chk1). In addition, HDACi reduce the expression of proteins involved in DNA repair (Rad51), mitotic spindle formation (TPX2) and chromosome segregation (Survivin) in glioma cells and in human glioblastoma multiforme primary cultures. Therefore, HDACi treatment causes glioma cell entry into mitosis before DNA damage could be repaired and to the formation of an aberrant mitotic spindle that results in glioma cell death through mitotic catastrophe-induced apoptosis.

Glioblastoma multiforme (GBM) is an incurable cancer due to its aggressiveness and its resistance to conventional anti-tumoral therapies. Multiple genetic alterations are involved in gliomagenesis leading to an aberrant activation of key pathways involved in mitogenic signaling and cell cycle control.^1,2^ The intratumoral heterogeneity combined with a putative cancer stem cell subpopulation underlies the difficulty to treat this cancer. The median survival of GBM patients treated with multimodal therapies including surgical resection, radiation and chemotherapy is less than 16 months due to tumor relapse after surgical removal.^3^

Histone deacetylases (HDAC) are key regulators of cell development and cancer, by deacetylating histones and other proteins.^4^ Recent studies found that class I HDAC expression was high in locally advanced, dedifferentiated and strongly proliferating tumors, sometimes associated with compromised patient prognosis.^5^ In contrast, a reduction in class II HDAC expression was described in different types of tumors, including GBM samples.^6^ Nevertheless, HDAC inhibitors cause the acetylation of both histone and non-histone proteins and exert multiple anti-tumoral effects by inducing differentiation, apoptosis, cell cycle arrest, susceptibility to chemotherapy and inhibition of migration and angiogenesis.^7^ Therefore, HDACi are widely investigated and tested as anticancer drugs. Initial clinical trials indicate that HDAC inhibitors from several structural classes are well tolerated and exhibit therapeutic activity against a variety of human malignancies, and the pleiotropic molecular mechanisms of action of these drugs are being uncovered.^8, 9, 10^ The elucidation of the key molecular targets of HDACi involved in glioma cell death is relevant for the development of more specific therapeutic strategies.

Here, we characterize the response of glioma cell lines and primary GBM cultures to two broad range HDACi being tested in clinical trials against GBM: suberanilohydroxamic acid (SAHA, vorinostat) and valproic acid (VPA). Both drugs are able to kill glioma cells more efficiently than the chemotherapeutic drug temozolomide (TMZ). We also present the analysis of the molecular alterations associated with glioma cell death, showing that HDACi drive cells to mitotic catastrophe and cell death by apoptosis.

## Results

### SAHA and VPA affect glioma cell viability, proliferation and clonogenicity

**Figure 1:**
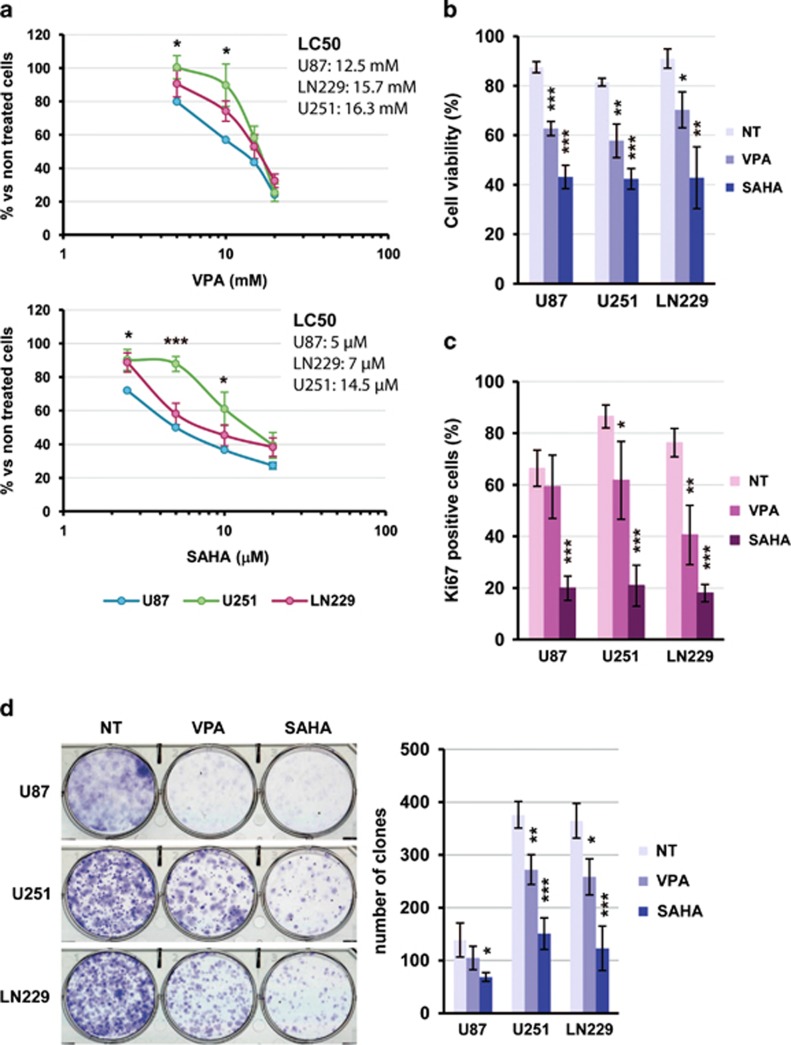
SAHA and VPA reduce glioma cell viability, proliferation and clonogenicity. (**a**) Dose−response of glioma cell lines U87-MG, U373-MG and LN229 to VPA (upper graph) and SAHA (lower graph) treatment by WST1- cell viability assay. Cells were treated for 48 h with increasing concentrations of HDACi. The LC50 for each cell line was estimated by interpolation in the line-graph. Data shown are mean±S.E.M. from five independent experiments. Statistical comparisons were performed against the most sensitive cell line, U87-MG. (**b**) Cell viability of glioma cell lines after 48- h treatment with 10 mM VPA, 10 *μ*M SAHA or left untreated. Cells were counted in a hemocytometer by trypan blue exclusion. Data are mean±S.E.M. from five independent experiments. (**c**) Analysis of cell proliferation by Ki-67-positive nuclei counting after 48 h of HDACi treatment (10 mM VPA or 10 *μ*M SAHA). Data in the graphic are expressed as mean±S.E.M. of four independent experiments. (**d**) Clonogenic assay of glioma cell lines after 48-h treatment with HDACi (see Materials and Methods for details). Representative images of the stained clones are shown on the left. Bar graph shows results obtained from four independent experiments (Mean±S.E.M.). Statistical analysis were performed by the Student's *T-*test being *P<0.05; **P<0.01; ***P<0.001

On WST-1 assays, SAHA and VPA decreased cell viability in a concentration-dependent manner (Figure 1a). Only at intermediate concentrations, differences between glioma cell lines were observed, being U251-MG cells less sensitive than U87-MG cells. LC50 values (Figure 1a) showed that U251-MG has the lower sensitivity to both HDACi. Similar results were obtained by viable cell counting using trypan blue exclusion at selected HDACi concentrations (Figure 1b), being 10 *μ*M SAHA more effective than 10 mM VPA.

Next, we analyzed the effect of VPA and SAHA on cell proliferation by Ki-67 immunodetection. Treatment with 10 *μ*M SAHA for 24 h reduced the percentage of proliferating cells from a range of 70–90% to less than 20% in all cell lines (Figure 1c), whereas 10 mM VPA had a weaker effect and only significant in U251-MG and LN229 cells. Clonogenic assays showed that HDACi reduced significantly the capability of cells to grow clonally after a 48-h treatment, (Figure 1d).

### HDACi promote nuclear condensation and the activation of the mitochondrial apoptotic pathway

**Figure 2:**
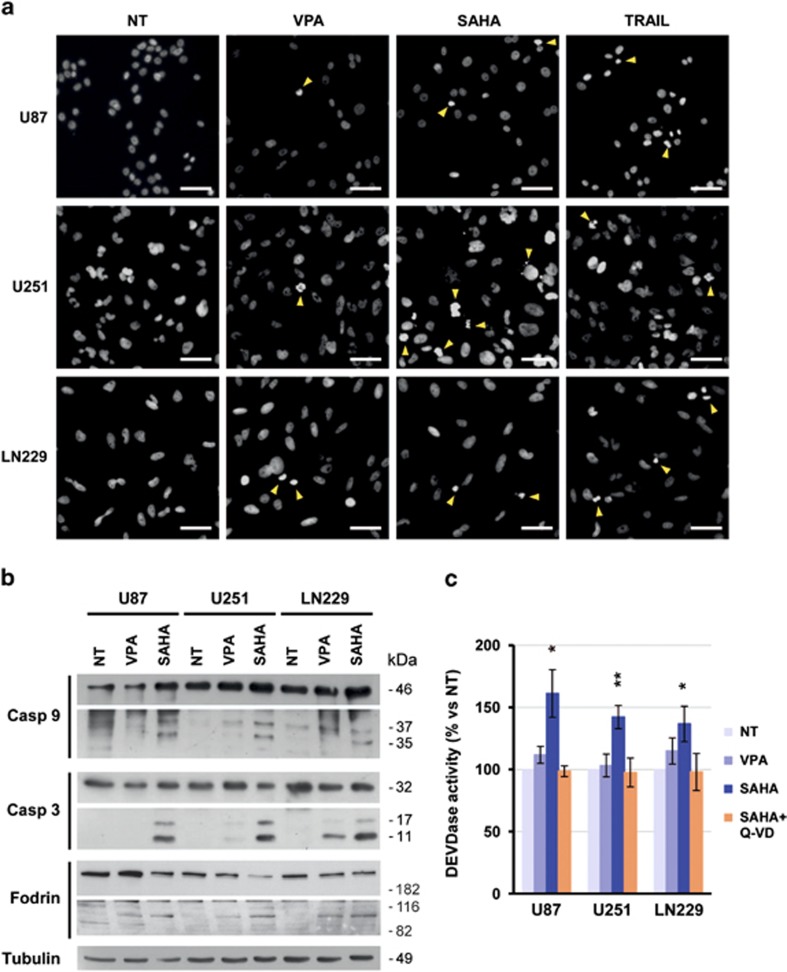
VPA and SAHA induce nuclear condensation and caspase activation in glioma cell lines. (**a**) Nuclear morphology of U87-MG, U373-MG and LN229 after 24-h treatment with 10 mM VPA, 10 *μ*M SAHA or 100 ng/ml TRAIL. After treatment, cells were fixed in 4% PFA and nuclei were stained with Hoechst 33342. Condensed and abnormal nuclei are indicated with yellow arrowheads. Scale bar= 50 *μ*m. (**b**) Analysis of caspase-9, caspase-3 and fodrin expression and cleavage on glioma cell lines after 24-h treatment with 10 mM VPA and 10 *μ*M SAHA by western blot. Membrane was reprobed with an anti-*α*-tubulin antibody to verify equal loading. A representative blot from three independent experiments is shown. (**c**) Caspase-3 activity in HDACi-treated cells. Glioma cells were treated with 10 mM VPA, 10 *μ*M SAHA alone or in combination with the caspase inhibitor Q-VD-OPh (5 *μ*M) for 24 h. Cell lysates were incubated with Ac-DEVD-AFC fluorogenic substrate up to 8 h, and its cleavage was measured hourly using a fluorometer. Bars depict mean±S.E.M. from four independent experiments. Statistical analysis was performed using the Student's *T*-test by comparing treated *versus* non-treated (NT) cells (*P<0.05, **P< 0.01)

Our results showed that SAHA and VPA decrease cell viability dose dependently. Therefore, to assess the participation of the apoptotic cell death pathway on this effect, we analyzed the nuclear morphology of glioma cells after 24 h of treatment. As a reference, apoptosis inducer TRAIL was added to cells at 100 ng/ml. Nuclear staining with Hoechst showed that HDACi cause nuclear condensation and abnormal nuclear morphology in some cells (Figure 2a). However, nuclear fragmentation was absent and it was very infrequent in TRAIL-treated cells. Therefore, glioma cells do not show a typical apoptotic nuclear fragmentation after VPA and SAHA treatment. In addition, HDACi induced an increase in nuclear size in U87-MG and LN229 cells, indicating that chromatin was remodeled due to histone acetylation (Supplementary Figure 1).

To determine whether HDACi induced the intrinsic apoptotic pathway, we checked the expression and cleavage of caspase 9 and caspase 3 by western blot (Figure 2b) and executioner caspase activity by a fluorogenic assay (Figure 2c). We observed that 10 *μ*M SAHA promoted the cleavage of both Caspase 9 and Caspase 3, whereas 10 mM VPA had a weaker effect in all glioma cell lines. As expected, Caspase-3 cleavage correlated with an increase in the DEVDase activity of SAHA-treated cells (Figure 2c). Therefore, HDACi, mainly SAHA at the concentration tested, are able to promote the activation of the mitochondrial apoptotic pathway in glioma cells. HDACi used in this study did not activate the extrinsic apoptosis pathway on glioma cells (unpublished results).

### HDACi promote DNA fragmentation in glioma cells, which is dependent on the activation of the caspase/CAD pathway

**Figure 3:**
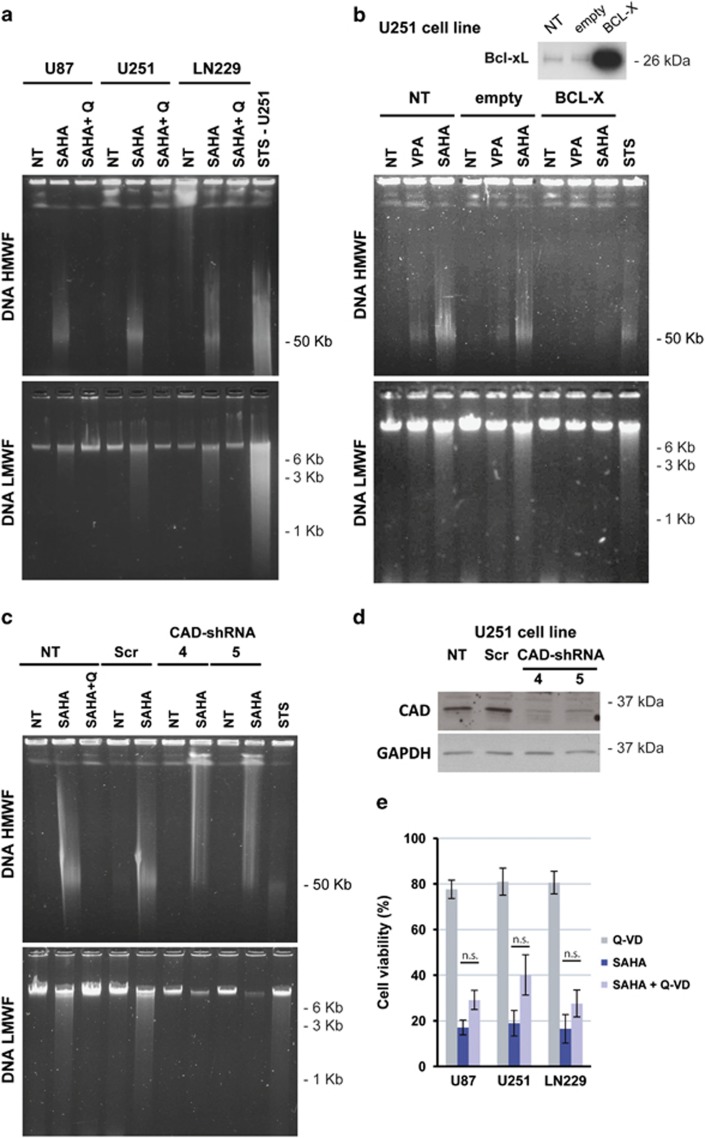
HDACi promote DNA fragmentacion in glioma cell lines, which is dependent on caspase activation. (**a**) DNA fragmentation analysis on glioma cells treated for 48 h with 10 *μ*M SAHA combined or not with the pan-caspase inhibitor Q-VD-OPh (5 *μ*M) by PFGE (HMWF, upper picture) and ladder (LMWF, lower picture) procedures. As a positive control, LN229 cells were treated with 1 *μ*M STS for 48 h. (**b**) Analysis of the effect of *BCL-X* overexpression on DNA fragmentation (HMWF and LMWF) induced by 10 mM VPA or 10 *μ*M SAHA on U251-MG glioma cells. Bcl-xL expression was checked by western blot (upper panel). (**c**) Downregulation of CAD expression with specific shRNA (4 and 5) blocks the formation of 50- Kb fragments and low-molecular weight fragmentation on U251-MG cells treated for 48 h with 10 *μ*M SAHA. (**d**) Western blot of CAD on U251-MG cells transduced with lentiviral vectors containing CAD shRNA or scrambled sequence (Scr). Equal loading was verified by GAPDH detection of the same membrane (lower panel). Each DNA integrity assay shown in **a**, **b** and **c** was repeated three times and a representative image is shown. (**e**) Cell viability analysis by cell counting using trypan blue exclusion on glioma cells treated with 10 *μ*M SAHA or 5 *μ*M Q-VD-OPh or both combined for 48 h. Bars depict mean±S.E.M. from four independent experiments. Statistical analysis was performed by the Student's *T-*test, no significant differences (n.s.) were obtained by comparing SAHA and SAHA+ Q-VD-treated cells

Next, we checked whether the activation of the caspase cascade by HDACi promoted DNA fragmentation. We analyzed DNA integrity by Pulsed-field gel electrophoresis (PFGE) and agarose gel electrophoresis (Figure 3a). SAHA treatment for 48 h caused the formation of 50- kb DNA fragments and the final DNA degradation in a smear pattern (Figure 3a). Interestingly, the addition of a pan-caspase inhibitor (Q-VD-OPh) blocked both high- and low-molecular weight DNA fragmentation, indicating that caspase activation was necessary for DNA degradation. Staurosporine (STS), an apoptosis inducer, was added for 24 h to U251-MG cells as a positive control for DNA fragmentation. The formation of 50- Kbp DNA fragments was observed, but typical oligonucleosomal laddering was not detected (Figure 3a). These results indicated that U251-MG cells exposed to an apoptosis inducer do not perform DNA internucleosomal fragmentation. To further corroborate the implication of the mitochondrial apoptotic pathway in SAHA-induced DNA degradation, we overexpressed the antiapoptotic gene *BCL-X* in U251-MG glioma cells (Figure 3b). We observed that Bcl-xL-overexpressing cells were protected against the induction of DNA degradation by SAHA, further suggesting the involvement of the intrinsic apoptotic pathway in SAHA effects.

One of the nucleases responsible for DNA processing on caspase activation is Caspase-associated DNAse (CAD).^11^ Therefore, we next verified whether CAD endonuclease was implicated in SAHA effects by using two shRNAs against CAD mRNA that efficiently downregulate CAD protein levels in U251-MG cells (Figure 3d). After SAHA treatment, we observed that CAD downregulation blocked the formation of 50-Kbp and low-molecular weight DNA fragments, leading to bigger DNA fragments showing a smear pattern on the upper part of the gel. This indicates that CAD is the nuclease implicated in the formation of 50-Kbp fragments on HDACi treatment and suggests that 50-Kbp DNA processing is required for the final low-molecular weight DNA degradation. As suggested by the smear pattern that appears in PFGE experiments when CAD expression is reduced (Figure 3c) but not when caspases are inhibited with Q-VD-OPh (Figure 3a) or on Bcl-xL overexpression (Figure 3b), other caspase-activated nucleases may participate in DNA initial processing; however, the identity of this nuclease/s remains to be investigated.

As caspases were involved in DNA fragmentation, we next checked the relevance of caspase activation in HDACi-induced glioma cell death (Figure 3e). Trypan blue exclusion cell counts showed that caspase inhibition did not prevent cell death, indicating that cells died even in the absence of caspase activation, thus indicating that the apoptotic pathway is not required for HDACi-induced glioma cell death.

### HDAC inhibition in glioma cells promotes DNA damage partly due to ROS production

**Figure 4:**
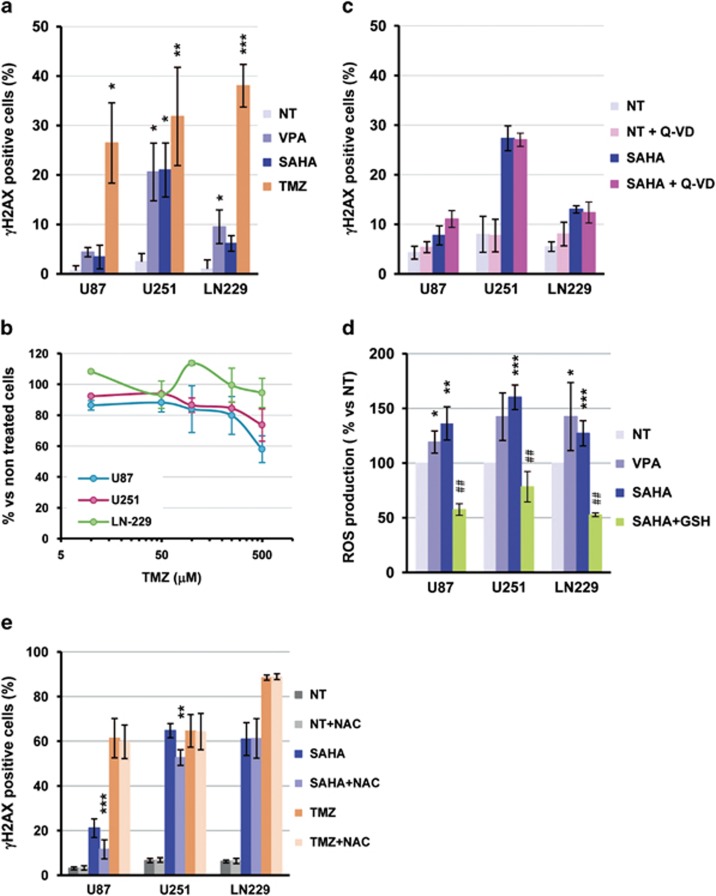
HDACi promote DNA damage by DSBs formation in glioma cell lines. (**a**) Analysis of DNA DSB formation by immunofluorescence of γH2AX-positive nuclei in glioma cells treated for 48 h with 10 mM VPA, 10 *μ*M SAHA or 100 *μ*M TMZ. Data are mean±S.E.M. from five independent experiments. (**b**) WST-1 cell viability assay on glioma cell lines exposed to increasing concentrations of TMZ (from 10  to 500 *μ*M) for 48 h. Data shown are mean±S.E.M. from three independent experiments. (**c**) Analysis of γH2AX-positive nuclei on glioma cells treated with 5 *μ*M Q-VD-OPh, 10 *μ*M SAHA or both combined for 48 h. Bars depict mean±S.E.M. from four independent experiments. (**d**) ROS production measured by flow cytometry on glioma cells treated with 10 mM VPA, 10 *μ*M SAHA or with 10 *μ*M SAHA in the presence of 10 mM GSH. Data are mean±S.E.M. from four independent experiments. (**e**) Analysis of γH2AX-positive nuclei on glioma cells treated with 10 *μ*M SAHA or 100 *μ*M TMZ in the presence or absence of 15 mM *N*-acetyl-*L*-cysteine (NAC). Bars depict mean±S.E.M. from four independent experiments. Statistical analysis was performed by the Student's *T*-Test, *P<0.05, **P< 0.01, ***P<0.001 in comparison with NT cells or ^##^*P*<0.01 in comparison with SAHA-treated cells

One of the mechanisms involved in the activation of the mitochondrial pathway is the activation of the DNA damage response. Therefore, we next verified whether SAHA and VPA were able to promote the formation of DNA double-strand breaks (DSBs). Cells treated with temozolomide (TMZ), an alkylating drug in current use for GBM treatment, were used as positive control (Figure 4a). SAHA and VPA promoted an increase in the percentage of γH2AX-positive cells, a marker of DSBs formation, and significant changes in γH2AX staining for both drugs were attained in U251-MG cells. As expected, TMZ induced a strong increase in γH2AX-positive nuclei in glioma cells.

As TMZ was more efficient in promoting DNA damage, we next verified the sensitivity of glioma cell lines to this drug. Surprisingly, all three cell lines showed a higher resistance to TMZ than to HDACi used in this study (Figure 4b compared with Figure 1a). In fact, at the maximal concentration of 500 *μ*M, TMZ did not even reach a 40% decrease in cell viability of the most sensitive cell line U87-MG compared with more than 70% attained by HDACi at the highest doses.

To determine the role of caspase activation in HDACi-induced DNA damage, we next analyzed the effect of caspase inhibition in γH2AX-positive cells after SAHA treatment (Figure 4c). Results obtained show that caspase inhibition with Q-VD-OPh did not block the formation of DSBs in genomic DNA, indicating that SAHA-induced DNA damage occurs independently of caspase activation.

It has been reported that HDAC inhibition produces an increase in reactive oxygen species (ROS) and that this could contribute to the promotion of DNA damage. We therefore analyzed ROS production in HDACi-treated cells (Figure 4d). Both HDACi tested caused a significant increase in ROS abundance, whereas TMZ did not have this effect. ROS production correlated with an increased protein carbonylation in SAHA-treated U87-MG cells (Supplementary Figure 2A). SAHA-induced ROS generation was blocked by glutathione (GSH) addition (Figure 4d). The mechanism involved in SAHA-induced ROS production in tumoral cells has been proposed to be by the upregulation of thioredoxin-binding-protein-2 (TBP-2) and by the downregulation of thioredoxin activity.^12^ We assessed the effect of both HDACi on TBP-2 and thioredoxin protein levels in our cell model, but we did not detect any change neither in TBP-2 nor in thioredoxin expression (Supplementary Figure 2B).

With the aim to determine whether ROS production is involved in HDACi-induced DNA damage, we checked *γ*H2AX staining on SAHA- and TMZ-treated cells in the presence or absence of ROS inhibitors. We observed that ROS scavenging with 15 mM NAC partially prevented DNA DSBs formation in U87-MG cells and in U251-MG cells, but not in LN229 cells (Figure 4e), pointing out to the existence of an additional molecular alteration that results in DSBs formation in addition to oxidative stress.

### HDACi affect cell cycle progression by decreasing G2 checkpoint kinase expression: Wee1 and Chk1

**Figure 5:**
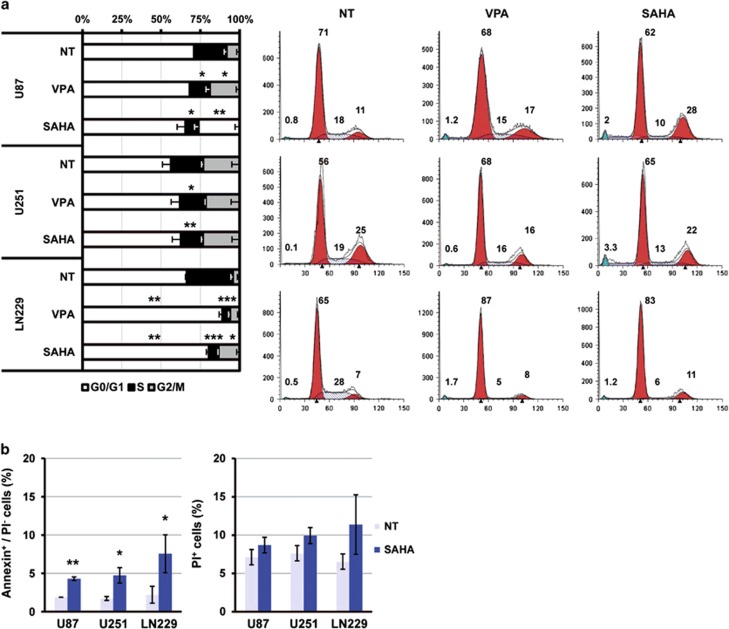
HDACi treatment of glioma cell lines alters cell cycle progression, decreasing the percentage of cells in S phase and increasing cell population in G2/M. (**a**) Cell cycle analysis by flow cytometry of glioma cell lines treated for 24 h with 10 mM VPA, 10 *μ*M SAHA or left untreated. Bar graph shows the mean of the percentage of cell in G0/G1, S and G2/M phases with error bars (S.E.M.) from three independent experiments. Plots on the right are representative results from one experiment. (**b**) Percentage of apoptotic cells (Annexin V positive and PI negative) and dead cells (PI positive) after 24 h of treatment. Results are mean±S.E.M. from four independent experiments. Statistical analysis was performed by the Student's *T*-test and significance is shown by *P<0.05; **P<0.01

To further characterize glioma cell response to SAHA and VPA, we next analyzed cell cycle distribution by flow cytometry. We observed that, after 24 h, HDACi promoted a significant decrease of U87-MG and LN299 cells in S phase and an increase of cells in G2/M (Figure 5a). Changes on U251-MG cells reached significance after 48h of incubation (data not shown). In addition, an increase of cells in G0/G1 phase was observed in LN229 cells. The reduction of cells in S phase can be due to the promotion of cell cycle arrest or a faster progression to the G2/M phase. However, only LN229 cells showed an arrest in G0/G1. The increase of G2/M-cell population at the expense of G0/G1 and S phases suggests an alteration on G2 checkpoint. Interestingly, subG1 peak increased in all glioma cell lines after 48 h of treatment (Supplementary Figure 3). Therefore, we next checked whether SAHA induced apoptosis in glioma cells, using Annexin V-Propidium Iodide (PI) double staining. Data presented on Figure 4e show that SAHA promoted an increase in the percentage of Annexin V-positive cells in the PI-negative population (conserved membrane integrity, left bar-graph), whereas no significant changes were observed in the PI-positive cell population, indicating phosphatidylserine exposure in the cell membrane. These results indicate that SAHA promotes apoptotic cell death in glioma cells.

**Figure 6:**
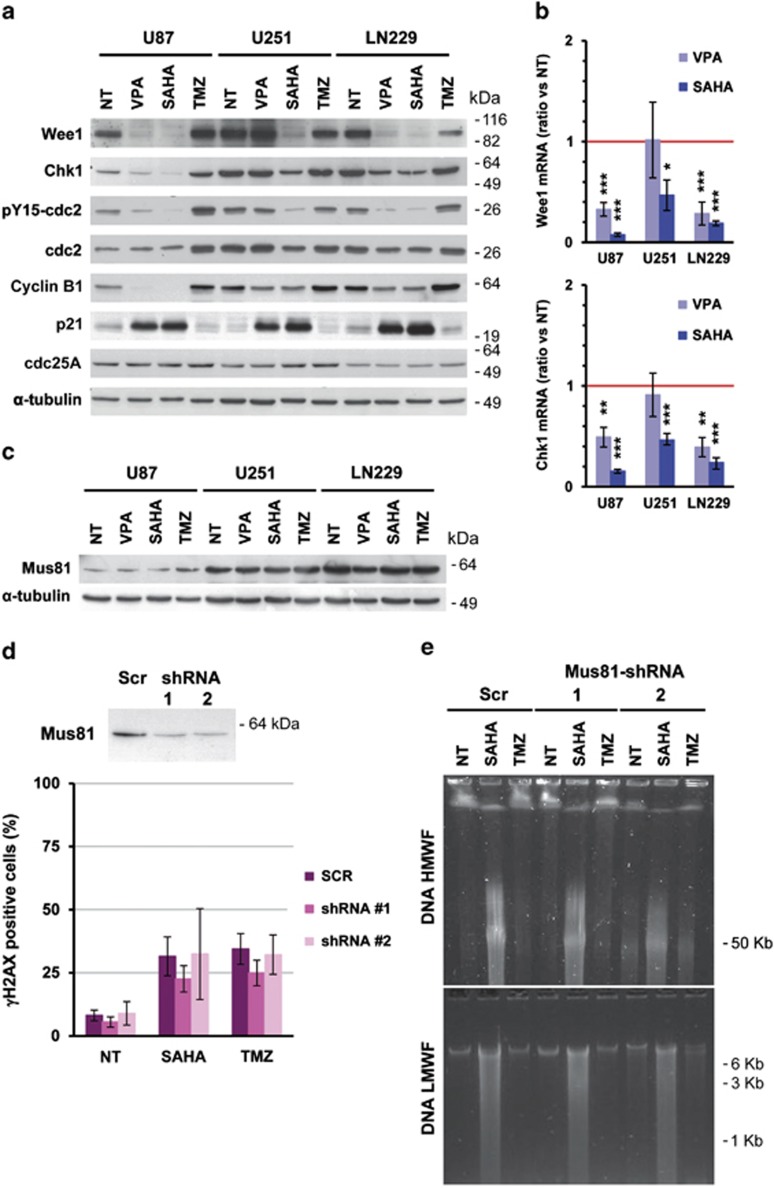
SAHA and VPA reduce the levels of expression of G2 checkpoint gatekeepers Wee1 and Chk1. (**a**) Analysis of the effect of HDACi on Wee1 and Chk1 protein expression and on the phosphorylation status of cdc2 (Tyr-15) in glioma cells. Cell cycle regulators, Cyclin B1, p21 and cdc25A, were also analyzed. Cells were treated for 24 h with 10 mM VPA, 10 *μ*M SAHA or 100 *μ*M TMZ, and protein extracts were analyzed by electrophoresis and western blot using specific antibodies against proteins indicated on the left of the panels. Representative blots of three independent experiments are shown. (**b**) Analysis of Wee1 and Chk1 mRNA after 24 h of HDACi treatment of glioma cell lines by reverse transcription and quantitative real-time PCR. Bars depict the mean±S.E.M. of four independent experiments. (**c**) Expression of Mus81 endonuclease in glioma cells treated as described above by western blot. Equal loading was verified by *α*-tubulin detection on the same membrane. (**d**) Downregulation of the endonuclease Mus81 do not prevent DSBs formation in SAHA-treated U251-MG cells. The efficacy of two specific shRNA against the mRNA of Mus81 (1 and 2) was verified by western blot (upper panel). Percentage of γH2AX-positive cells after 48 h of incubation in the presence of 10 *μ*M SAHA or 100 *μ*M TMZ of lentivirus-transduced cells. Data are mean±S.E.M. from three independent experiments. (**e**) Analysis of DNA fragmentation after Mus81 downregulation with shRNA. Representative gels of three independent experiments are shown, of high-molecular weight DNA fragmentation (upper panel) and low-molecular weight DNA fragmentation (lower panel) analysis

The accumulation of cells in G2/M prompted us to check whether HDACi affect the expression of G2 checkpoint kinases: Chk1 and Wee1. We observed that 10 *μ*M SAHA caused a strong decrease in Chk1 and Wee1 protein abundance (Figure 6a), correlating with a decrease in cdc2 phosphorylation on Tyr-15, which is the final effector for M-phase progression and Wee1 substrate. VPA treatment had a weaker effect and also reduced the amount of Chk1 and Wee1 in U87-MG and LN229 cells, respectively. In contrast, the treatment with TMZ, which directly causes DNA damage, did not affect the amount of Chk1 and Wee1 kinases. Interestingly, the more resistant cell line U251-MG expresses higher levels of Wee1, followed by LN229 cells and the U87-MG cells. Chk1 was also more expressed in U251-MG and LN229 cells than in U87-MG cells. Results obtained by western blot agreed with Wee1 and Chk1 mRNA levels measured by quantitative real-time RT-PCR (Figure 6b), indicating that HDACi affect Wee1 and Chk1 expression at the transcriptional level.

As the expression of cdc2 was not affected by HDACi treatment, but the inhibitory phosphorylation on Tyr-15 diminished, we next checked the expression of its cyclin partner (Figure 6a). We observed that cyclin B1 decreased after HDACi treatment and the expression of the cell cycle inhibitor p21 was strongly increased concurring with published data.^13^ In contrast, the amounts of cdc25A, a phosphatase involved in the regulation of G1/S and G2/M checkpoints,^14^ were unaffected, suggesting that cells were not arrested through a decrease in cdc25A. As cdc25A is regulated post-translationaly and by subcellular localization, a more detailed characterization of other cell cycle regulators in synchronized cells would be necessary to confirm whether cell cycle arrest occurs on a subpopulation.

Wee1 inhibition has been reported to increase replication stalling and DSBs induced formation by the endonuclease Mus81.^15,16^ Therefore, we checked whether Mus81 is implicated in HDACi-induced DNA damage. As shown in Figure 6c, none of the drugs altered the expression of Mus81. Moreover, Mus81 downregulation by shRNA did not reduce SAHA-induced DSBs formation nor low- and high-molecular weight DNA fragmentation either (Figures 6d and e), discarding the implication of this endonuclease in SAHA DNA-damaging effects.

### HDACi cause glioma cell death by promoting mitotic catastrophe

**Figure 7:**
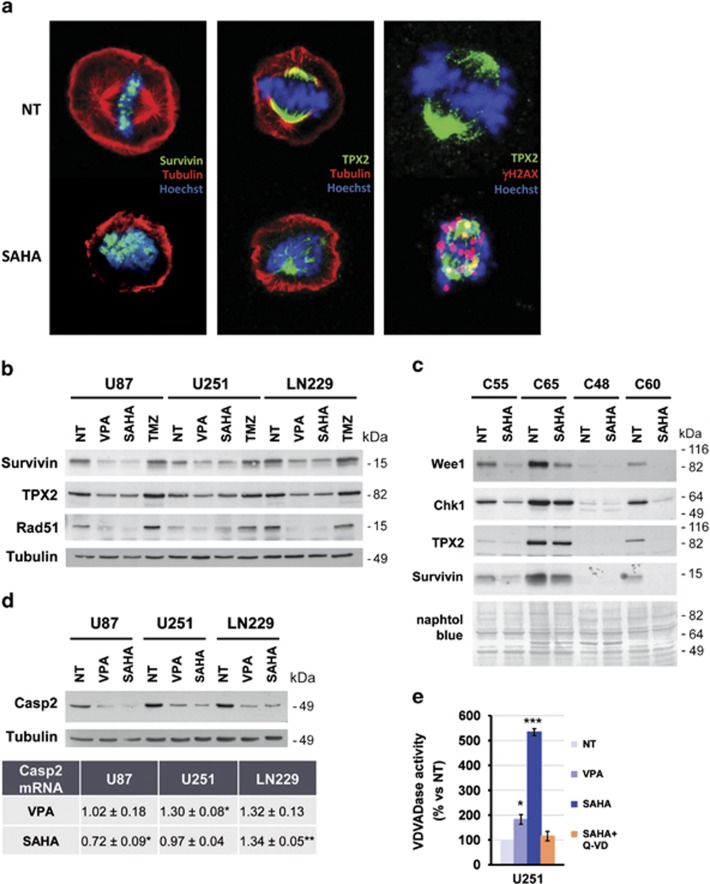
HDACi promote cell death by mitotic catastrophe. (**a**) Analysis of U251-MG cells in metaphase after SAHA treatment by immunofluorescence of Survivin (in green, left panel) and TPX2 (in green, middle panel), *α*-tubulin (in red, left and middle panels), γH2AX (in red, right panel) and nuclear staining with Hoescht 33342 (in blue, all panels). Images were acquired using an Olympus IX-70 confocal microscope and representative pictures from three independent experiments are shown. (**b**) SAHA and VPA reduce the expression levels of Rad51, Survivin and TPX2 in glioma cell lines. Total cell lysates from glioma cells were analyzed by electrophoresis and western blot using specific antibodies against the proteins indicated on the left. Representative blots from three independent experiments are shown. (**c**) Analysis of the effect of SAHA on Wee1, Chk1, TPX2 and Survivin protein expression in human GBM primary cultures. Representative blots are shown from three independent experiments. Equal loading was checked by membrane staining with naphtol blue. (**d**) Caspase 2 protein and mRNA expression in HDACi-treated glioma cell lines. After a 24-h treatment with 10 mM VPA or 10 *μ*M SAHA, cells were processed for western blot analysis of Caspase-2 (upper panel) or RNA was extracted to perform quantitative RT-PCR (lower table). Data are shown as a ratio *versus* non-treated cells, and the mean±S.E.M. from three independent experiments is summarized. Statistical analysis were performed by the Student's *T-*test being *P<0.05; **P<0.01. (**e**) Caspase-2 activity assay on U251-MG cells treated for 24 h with 10 mM VPA, 10 *μ*M SAHA in the presence or absence of 5 *μ*M Q-VD-OPh. Cell lysates were incubated with Ac-VDVAD-AFC fluorogenic substrate up to 8 h and its cleavage was measured hourly using a fluorometer. Bars depict mean±S.E.M. from three independent experiments. Student's *T*-test was performed by comparing data from treated *versus* non-treated (NT) cells (*P<0.05, ***P< 0.001)

The fact that HDACi cause a decrease in the expression levels of Wee1 and Chk1 made us to hypothesize that cell death may occur through mitotic catastrophe due to the accumulation of DNA damage that cannot be repaired before mitosis.^17,18^ Therefore, we next analyzed by confocal microscopy the nuclear morphology of cells in division after SAHA treatment. Untreated cells had a normal distribution of chromosomes in metaphase, and mitotic spindles were correctly formed (Figure 7a). However, on SAHA treatment dividing cells showed aberrant metaphase morphologies: chromosomes were not aligned at the metaphase plate, Survivin was not recruited to centromeres and an abnormal distribution of mitotic spindle was observed (Figure 7a). Aberrant mitotic figures were formed, and no cells in telophase or cytokinesis were observed, indicating that cells cannot complete mitosis leading to cell death. In addition we observed that cells enter mitosis with damaged DNA, as chromosomes from SAHA-treated cells were labeled with the γH2AX antibody (Figure 7a, right image). In addition, HDACi promoted a decrease in the amount of Survivin and TPX2 (Figure 7b), indicating a deficit of proteins relevant for correct mitosis completion. In addition, we determined the expression of Rad51, a protein involved in homology-directed repair of DNA DSBs. Interestingly, HDACi also reduced the expression levels of Rad51 in glioma cell lines in comparison with untreated cells and TMZ-treated cells (Figure 7a). Therefore, this indicates that HDACi impair DNA repair mechanisms in glioma cells, as it has been described in other types of tumor cells.^19^

As HDACi induced a strong decrease of key proteins involved in cell cycle control and mitosis, we verified whether these effects were extensive to primary GBM cultures. Cells isolated from human GBM tumors were treated with 10 *μ*M SAHA for 24 h (Figure 7c). Three of the four primary GBM cultures reduced the expression of Wee1, Chk1, Survivin and TPX2 after SAHA treatment. Only cells of GBM C48, which expressed very low levels of these proteins in basal conditions, did not show the same response. These results suggest that HDACi have the same molecular effects on glioma cells independently of their origin and genetic alterations, and validate the results obtained in glioma cell lines. Therefore, HDACi alter the expression of proteins necessary for tumoral cell proliferation, which have been previously proven to be involved in gliomagenesis.

Together, these results indicate that glioma cells treated with SAHA die during mitosis by a mechanism involving mitotic catastrophe. One of the initial events that have been reported during this type of cell death is the activation of Caspase-2, upstream of cytochrome c release.^20^ Therefore, we checked Caspase-2 activation after HDACi treatment (Figure 7d). We observed that HDACi caused a decrease of procaspase-2 (51 kDa) protein, but no cleaved forms were detected (p32 or p18). However, Caspase 2 mRNA levels were unchanged in U251-MG and LN229 cells after HDACi treatment (table in Figure 7d). Measurement of caspase-2 activity in U251-MG cell lysates confirmed that VPA and SAHA promoted the activation of caspase 2, and that Q-VD-OPh addition blocked its activation (Figure 7e). Therefore, our results demonstrate the implication of Caspase 2 in glioma cell death induced by HDACi.

## Discussion

Our aim was to characterize the molecular changes induced by broad-range HDAC inhibitors that are used for killing chemotherapy-resistant glioma cells. Here we have demonstrated that two structurally different HDACi, SAHA and VPA, have multiple effects in glioma cancer cells that result in oxidative stress, DNA damage and the activation of the apoptotic mitochondrial pathway. However, the HDACi-induced apoptotic pathway is not the main cause of glioma cell death. Rather, HDACi treatment impairs G2 checkpoint, pushes glioma cells to enter mitosis before DNA damage is repaired and decreases the expression of proteins that have crucial roles in mitosis, thus triggering cell death by mitotic catastrophe.

HDAC inhibition in glioma cell lines causes cell death in a dose-dependent manner, whereas those cells show resistance to the chemotherapeutic agent TMZ. The resistance of GBM cells to chemo- and radiotherapy has recently been attributed to the overexpression of the G2 checkpoint kinase Wee1.^18^ We have shown that HDAC inhibition alters the expression of two G2 checkpoint kinases, Wee1 and Chk1, and results in cells entering mitosis before DNA-damage repair. In accordance with our results, pharmacologic inhibition of G2 checkpoint kinases Wee1 and Chk1 has been shown to have anti-tumoral effects in neuroblastoma cells both *in vitro* and *in vivo* ^21^ and cause DNA double-strand breaks in cancer cells in the absence of DNA-damaging chemotherapeutic drugs.^22^ In addition, Wee1 inhibition sensitizes GBM cells to ionizing radiation and pushes cancer cells onto mitotic catastrophe.^18^ In contrast, Chk1 inhibition in combination with a DNA-damaging drug, gemcitabine, drives rapid chromosome fragmentation followed by caspase-independent cell death.^17^ In fact, Chk1 has been demonstrated to control mitotic DNA damage checkpoint by delaying mitotic exit of DNA-damaged cells by regulating mitotic catastrophe.^23^ The role of Chk1 downregulation in HDACi-induced cell death has been previously demonstrated by Brazelle *et al.*^24^ in non-small cell lung cancer cells. Nevertheless here we describe for the first time the implication of Wee1 kinase in HDACi effects. Our results demonstrate that G2 checkpoint kinases are molecular mediators of HDACi-induced cell death on GBM cells and highlight the relevance of the administration of multitarget therapies for the treatment of resistant tumors.

Thioredoxin inactivation by the induction of thioredoxin-binding protein (TBP-2) has been previously reported to be important for HDACi-induced ROS production and DNA damage in different cancer cell types.^10,25,26^ However, we have not detected changes in TBP-2 expression, thus discarding this mechanism in our model. In fact, ROS production was implicated in DNA damage observed in U87-MG and U251-MG cells, but not in LN229, indicating that other molecular alterations may participate, such as alteration of the DNA-repair machinery,^19^ the acetylation of histones involved in DNA repair such as Histone 4^27^ or histone 3^28^ and the decrease in G2 checkpoint kinases.^22^ Our results show that HDACi display at least two of these effects on glioma cells, as they decrease the expression of G2 checkpoint kinases Wee1 and Chk1 and DNA damage repair protein Rad51. As the endonuclease Mus81 has been implicated in the DNA damage response induced by Wee1 depletion^16^ and in Chk1-deficient cells,^29^ we also checked whether Mus81 was a mediator of DSB formation and DNA fragmentation in SAHA-treated cells. However, our results indicate that Mus81 does not participate in the DNA-damaging effects of SAHA.

HDACi are able to activate the mitochondrial caspase-dependent apoptosis pathway in glioma cells. However, our results indicate that this event does not determine glioma cell death, as caspase inhibition does not prevent it. The detailed analysis of cell cycle distribution and nuclear morphology rather indicates that HDACi promote cell death by mitotic catastrophe due to an aberrant mitotic spindle formation, and the detection of caspase-2 activation supports this conclusion. In accordance with our results, a previous work described that HDACi treatment temporarily delays mitotic progression affecting prometaphase due to activation of the spindle assembly checkpoint, involving aberrant chromosome segregation and failed cytokinesis.^30^ Similarly, Trichostatin A has been shown to induce a delay at the G2/M transition, chromosome missegregation and multi-nucleation, and thereby it leads to cell death by promoting exit from aberrant mitosis without spindle checkpoint.^31^ Our results provide an explanation to these observations, as they show that HDACi significantly reduce the expression of G2 gatekeepers Wee1 and Chk1, in addition to other proteins involved in chromosome segregation, DNA repair and mitotic spindle formation. Therefore, HDACi-treated glioma cells die by a mechanism of mitotic catastrophe involving the activation of caspase-2 and the mitochondrial pathway due to DNA-damage accumulation and G2 checkpoint impairment, according to the current concept of mitotic catastrophe.^32^

Our results show that the expression of Rad51, a protein involved in DNA repair by homologous recombination, is reduced in HDACi-treated glioma cells. The same effect was described for the HDACi PCI-24781.^33^ In fact, it has been reported that SAHA affects the expression of proteins involved in several mechanisms of DNA repair: homologous recombination, non-homologous end joining or base excision repair^19^ in different kinds of tumor cells, such as RAD50 and MRE11 in prostate and lung cancer cells,^10^ Ku70, Ku80 and RAD50 in melanoma cells^34^ and Rad51 and BRCA1 in prostate cancer cells.^35^ HDACi-induced impairment of the DNA repairing potential could explain why antioxidant treatment does not prevent DSB formation in LN229 glioma cells. This implies that HDACi effects on proteins involved in DNA-repair are common and can contribute importantly to DNA damage, in conjunction with ROS production.

Our results also show that Survivin, an antiapoptotic protein that has been shown to be important for the regulation of mitosis,^36^ is downregulated by HDACi treatment in glioma cells. Survivin is overexpressed in most cancers and it can be used as a prognostic factor for several kinds of tumors.^37^ Therefore, therapies designed to downregulate or block Survivin have been experimentally tested. A dominant-negative mutant of Survivin significantly induced mitotic catastrophe and apoptosis, and inhibited tumor growth in a colon cancer xenograft model.^38^ It has also been described that cell division in the absence of Survivin results in defects in chromosome alignment, failure of cytokinesis and eventually cell death.^39^ Recently, it has been shown that the disruption of the Survivin-Ran complex by a pharmacological inhibitor abolishes survival and growth of glioma stem cells.^40^ Therefore, our findings together with previous data point out to the relevance of Survivin in cancer cell proliferation and support the role of this protein as a mediator of HDACi-induced mitotic catastrophe in glioma cells.

In conclusion, our results show that SAHA and VPA, which are more efficient than the chemotherapeutic drug TMZ in killing glioma cells, reduce the expression of proteins involved in processes relevant for tumor growth and survival: G2/M checkpoint gatekeepers, DNA repair mechanisms and proteins involved in mitotic spindle formation and chromosome segregation during mitosis, thus leading to cell death by mitotic catastrophe. These findings support the view that drugs targeting multiple processes are more efficient in killing cancer cells than DNA-damaging agents.

## Materials and Methods

### Materials and cell culture

Cell culture media, supplements and sera were from Gibco Life Technologies (Carlsbad, CA, USA). Temozolomide (3,4-dihydro-3-methyl-4-oxoimidazo-[5,1-d]-1,2,3,5-tetrazine-8-carboxamide, TMZ), valproic acid (VPA), SAHA (suberanilohydroxamic acid, vorinostat), L- reduced glutathione (GSH) and N-acetyl-L-cysteine (NAC) were purchased from Sigma-Aldrich (St. Louis, MO, USA). The TNF-related apoptosis-inducing ligand (TRAIL) was acquired from BioTrend Chemikalien GmbH (Köln, Cologne, Germany). Q-VD-OPh was purchased from Calbiochem (Merck KGaA, Darmstadt, Germany).

#### Glioma cell lines

U87-MG and U251-MG cells were obtained from CLS Cell Lines Service (Eppelheim, Germany), and LN229 cell line was from ATCC (Manassas, VA, USA). Cells were maintained in culture as indicated by the supplier at 37 °C and 5% CO_2_ atmosphere and were subcultured by trypsinization twice a week.

#### Primary cultures

Tumor biopsies were obtained from Hospital Universitari Arnau de Vilanova (HUAV; Lleida) and processed following the procedure described by Dr Seoane group.^41^

### Lentiviral Construction and Transduction

Human *BCL-X* cDNA was cloned into the expression lentiviral vector pEIGW.^42^ Primers for small hairpin RNA interference (shRNA) and control plasmid DNA pLKO.1-puro-SHC002 were bought to Sigma (hMus81-1: TRCN00000049727; hMus81-2: TRCN00000290878). Human *CAD* shRNA were kindly provided by Dr. Victor Yuste (UAB, Barcelona).^43^ Viruses were prepared and titered as described in Bahi *et al.*^44^ Cells were usually treated after 2–3 days of viral transduction when expression vectors were used or after 4–5 days when shRNA experiments were performed.

### WST-1 viability assay

Cell viability was measured using a colorimetric assay with WST-1 reagent (Roche Diagnostics GmbH, Mannheim, Germany). Glioma cells were seeded in 96-well plates (2–4 × 10^3^ cells/well), and, after 24 h, cells were treated for 48 h as indicated in the figures. WST-1 reagent was added and absorbance at 450 nm and 600 nm was measured in a BioTek plate reader (BioTek Instruments, Winooski, VT, USA) every hour up to 3 h. Cell viability was calculated as percentage of absorbance readings of treated *versus* untreated cultures.

### Clonogenic assay

Cells were plated in six-well plates at a density of 3.5–7 × 10^5^ cells/well. Once cells reached 70% confluency, HDACi were added and incubated for 48 h. Afterward, cells were trypsinized, counted by trypan blue exclusion and re-plated (500 cells/well) in duplicate in six-well plates. Cells were grown at 37 °C and 5% CO_2_ in normal medium and left to form colonies for 10 days, with a mid-term medium replacement. At the end, cells were fixed with cold-methanol for 10 min and stained with 0.5% crystal violet solution (Sigma) for 10 min at room temperature. Plates were rinsed carefully in water and left to dry overnight at room temperature. Finally, plates were scanned and clones were counted.

### Flow cytometry

#### Cell cycle analysis

Cells were seeded in 60-mm petri dishes at 1.25–2.5 × 10^6^ cells/dish, left to adhere and treated as indicated for 24 h. Afterward, cells were trypsinized and washed in ice-cold PBS, fixed in 70% ethanol and stored at –20 °C until analysis. Fixed cells were suspended in 500 *μ*l propidium iodide (PI)/ RNase staining buffer (Becton Dickinson, Franklin Lakes, NJ, USA), incubated for 30 min at 37 °C and analyzed in a BD FACSCanto II cytometer (Becton Dickinson). Data analysis was performed using ModFit LT software (Verity software house, Topsham, ME, USA).

#### Measure of ROS

Cells were plated in 35-mm dishes at 3.5 or 7 × 10^5^ cells/dish, left to adhere for 1 day and treated with the indicated drugs for 24 h at 37 ºC and 5%CO_2_. At the end Cell Rox Green Reagent (Life Technologies) was added to a final concentration of 2.5*μ*M and incubated for 30 min at 37 ºC. Cells were washed twice in cold PBS, trypsinized and suspended in 1ml PBS. Cells were analyzed in a BD FACSCanto II cytometer (Becton Dickinson).

#### Annexin V and propidium iodide staining

After 24 h of treatment with 10 *μ*M SAHA, cells were collected and incubated with 1 *μ*g/ml Annexin V-FITC (ENZO Life Sciences, Farmingdale, NY, USA) and 2.5 *μ*g/ml PI (BD Transduction Laboratories, Becton Dickinson, Franklin Lakes, NJ, USA) in 200 *μ*l buffer containing 10 mM Hepes/KOH (pH 7.4), 140 mM NaCl and 2.5 mM CaCl_2_ for 30 min at room temperature, and the cells were analyzed using a BD FACSCanto II cytometer (Becton Dickinson).

### Western blot and Immunofluorescence

Antibodies were obtained from commercial sources: Chk-1, MGMT, CAD, γ-Histone-2-AX (γH2AX), Ki-67, and fodrin from Millipore (Billerica, MA, USA); Wee1, Caspase-3 and Caspase-9 from Cell Signaling Technology (Danvers, MA, USA); *α*-tubulin from Sigma; Cdk1, Cdk1-PY15, GAPDH and Cyclin B1 from Abcam (Cambridge, UK); Mus81 from Thermo Scientific (Waltham, MA, USA); Survivin, TPX2, Rad51 from Novus Biologicals (Cambridge, UK); p21 and cdc25A from Santa Cruz Biotechnology (Dallas, TX, USA). Protein cell extracts and western blot was performed as previously described.^44^ Blots were developed using the enhanced chemiluminescence (ECL) or Super Signal reagents (Thermo Scientific). Membranes were reprobed with anti-*α*-tubulin or anti-glyceraldehyde-3-phosphate dehydrogenase (GAPDH) antibodies, or stained with napthol blue black dye (Sigma) for loading control.

For immunofluorescence experiments, glioma cells were grown on glass coverslips of 12 mm diameter and treated as described. Cells were fixed with 4% paraformaldehyde in PBS for 20 min at room temperature, blocked in 5% bovine serum albumin (BSA), 5% fetal bovine serum, 0.1% Triton X-100 in PBS for 1h  and incubated for 1h  at RT or overnight at 4 °C with primary antibodies. Samples were then incubated with secondary antibodies for 1h , stained with Hoechst 33342 (Molecular Probes Life Technologies) and mounted using Vectashield (Vector Laboratories, Peterborough, UK) or Mowiol (Sigma) mounting medium. Secondary antibodies used were anti-rabbit IgG-Alexa Fluor 488 and anti-mouse IgG-Alexa Fluor 594 (Molecular Probes Life Technologies).

### Quantitative RT-PCR

Total RNA was isolated from glioma cell lines after 24 h of treatment using the RNeasy kit Qiagen (Hilden, Germany) and quantified in a Nanodrop spectrophotometer (Wilmington, USA). Quantitative RT-PCR was performed using TaqMan Gene expression assays (Chk1, Wee1, Caspase 2 and GAPDH) and TaqMan Gene expression Master Mix (Life technologies) as previously described.^45^

### DNA integrity assay

Aliquots of treated and non-treated cells were collected by cell scraping, and pellets were frozen at –80 ºC. DNA integrity was checked by pulse-field gel electrophoresis (PFGE) and low-molecular weight fragmentation by agarose gel electrophoresis following a previously described procedure.^44^

### Caspase activity assays

Caspase activity was measured as previously described.^46^ Cells were treated with 10 mM VPA or 10 *μ*M SAHA for 24 h, in the presence or absence of 5 *μ*M Q-VD-OPh. Cells were lysed in a buffer containing 20 mM HEPES/NaOH (pH 7,2), 5 mM MgCl_2_, 1%PMSF, 1% Triton, 5 mM EDTA and 10 mM DTT, and protein concentration in the lysates was measured by the DC Protein assay (BioRad, Hercules, CA, USA). Equal loads of protein were added in 96-well plates and mixed with 50 mM of fluorogenic substrates: Ac-DEVD-AFC (Millipore, Billerica, MA, USA) for Caspase-3-like activity or Ac-VDVAD-AFC (ENZO Life Sciences, Farmingdale, NY, USA) for Caspase-2 activity, both at a final concentration of 50 *μ*M in 96-well plates. Assay plates were incubated at 37 °C, and fluorescence was read at intervals of 1 h during the next 8 h in a BioTek FL 600 fluorimeter (BioTek Instruments,), with excitation filter set at 360 nm or 400 nm and emission filter at 530 nm or 505 nm for Caspase-3 or Caspase-2, respectively. Data obtained for different experimental conditions were compared within the linear phase of absorbance increase. Data are mean of four independent experiments.

## Abbreviations

Bcl-xL: B-cell lymphoma-extra large protein
CAD: caspase-associated DNAse
DSBs: DNA double-strand breaks
GBM: glioblastoma multiforme
GSH: reduced glutathione
γH2AX: phosphorylated H2AX
HDAC: histone deacetylases
HDACi: histone deacetylase inhibitors
NAC: *N*-acetyl-*L*-cysteine
PFGE: pulsed-field gel electrophoresis
SAHA: suberanilohydroxamic acid
ROS: reactive oxygen species
TBP-2: thioredoxin-binding-protein-2
TMZ: temozolomide
TPX2: targeting protein for the Xenopus kinesin-like protein 2
VPA: valproic acid
