# Supplementary material for: Histone deacetylase inhibitors promote glioma cell death by G2 checkpoint abrogation leading to mitotic catastrophe
Source: Cell Death Dis. 2014 Oct 2;5(10):e1435–. doi: 10.1038/cddis.2014.412 (PMC4237242; doi:10.1038/cddis.2014.412)
Supplement: Supplementary Figure 1 [file cddis2014412x1.pdf]

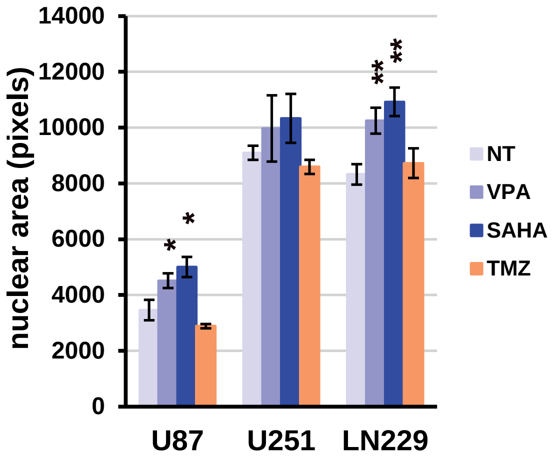

Supplementary Figure 1.

HDACi treatment of glioma cells cause an increase in the size of the nucleus. Glioma cells were treated with 10 mM VPA, 10 μM SAHA, 100 μM TMZ or left untreated. Nuclear area was measured on fluorescence pictures of Hoechst stained cells by using the ImageJ software (Rasband, W.S., ImageJ, U. S. National Institutes of Health, Bethesda, Maryland, USA, http://imagej.nih.gov/ij/, 1997-2014). Data represent Mean and S.E.M of four independent experiments. Statistical analysis was performed by the Student T-test and significance is represented by *p<0.05, **p<0.01,
