# Supplementary material for: Histone deacetylase inhibitors promote glioma cell death by G2 checkpoint abrogation leading to mitotic catastrophe
Source: Cell Death Dis. 2014 Oct 2;5(10):e1435–. doi: 10.1038/cddis.2014.412 (PMC4237242; doi:10.1038/cddis.2014.412)
Supplement: Supplementary Figure 2 [file cddis2014412x2.pdf]

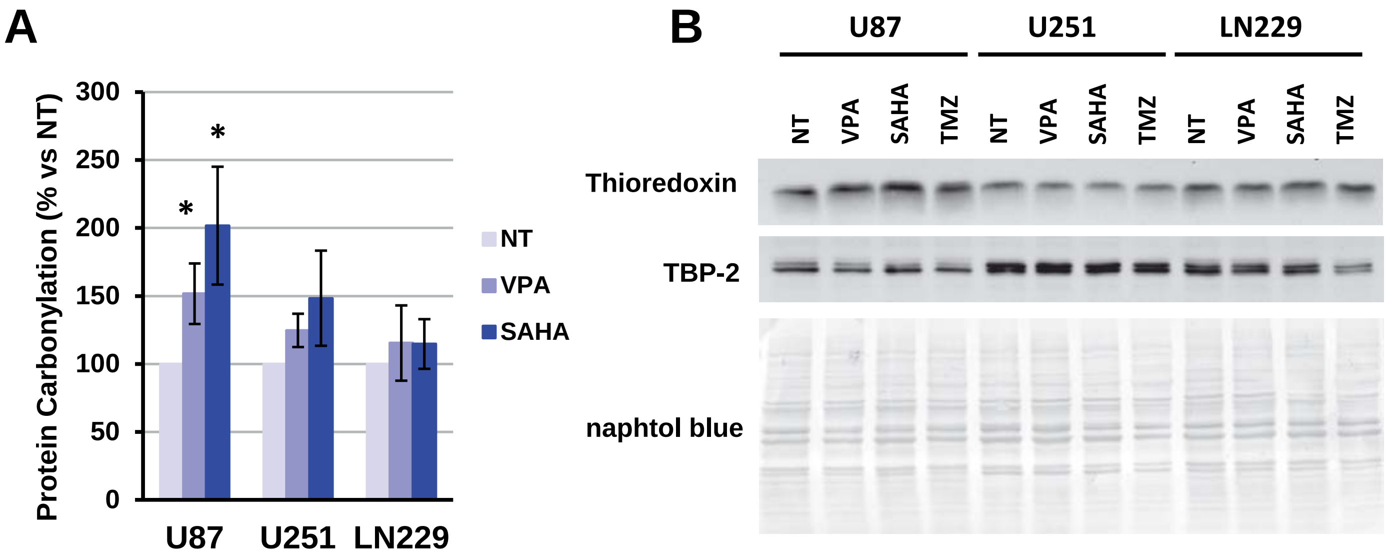

Supplementary Figure 2.

A. Total protein carbonylation quantified from western blots on total cell lysates. Cells were treated with 10 mM VPA, 10 µM SAHA or 100 µM TMZ for 24 hours. Results shown are Mean and SEM from 4 independent experiments. B. Analysis of the effect of HDACi on Thioredoxin and Thioredoxin binding protein 2 (TBP-2) protein expression in glioma cells. After 24h-treatment with 10 mM VPA, 10 µM SAHA or 100 µM TMZ, cells were lysed and analyzed by electrophoresis and western blot using specific antibodies from ThermoFisher Scientific against Thioredoxin 1 (MA5-1494) and TBP-2 (PA5-23485). Equal loading was verified by naphtol blue membrane staining (lower panel).
