# Supplementary material for: Histone deacetylase inhibitors promote glioma cell death by G2 checkpoint abrogation leading to mitotic catastrophe
Source: Cell Death Dis. 2014 Oct 2;5(10):e1435–. doi: 10.1038/cddis.2014.412 (PMC4237242; doi:10.1038/cddis.2014.412)
Supplement: Supplementary Figure 3 [file cddis2014412x3.pdf]

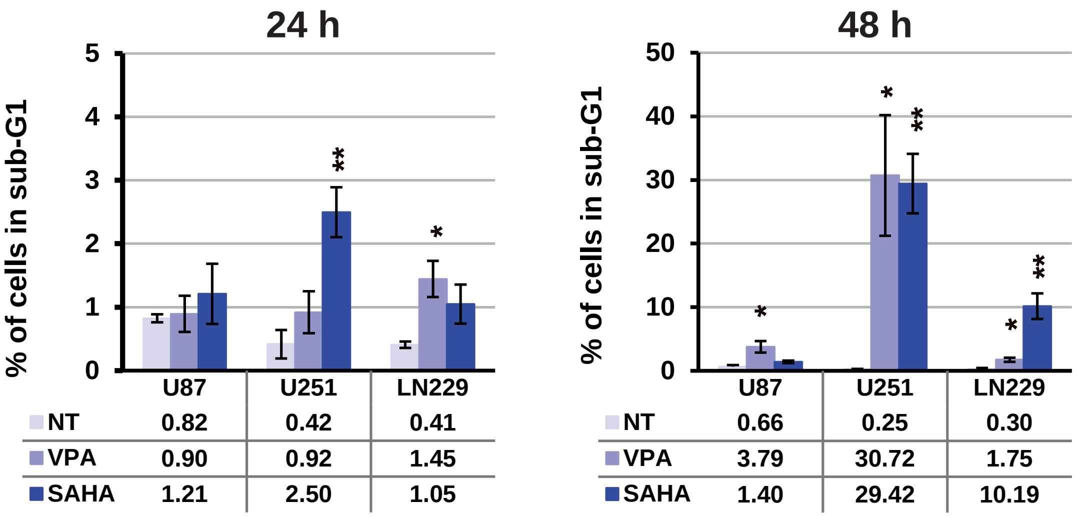

Supplementary Figure 3
HDACi treatment of glioma cells cause an increase in the amount of cells in sub-G1 by cell cycle analysis. Percentage of cells in sub-G1 by flow cytometry analysis of DNA content after 24h (left graph) or 48h (right graph) of treatment. Glioma cells were treated for 24 h and 48h with 10 mM VPA, 10 μM SAHA or left untreated. Results are mean and S.E.M. from three independent experiments. Statistical anlysis was performed by the Student T-test and significance is represented by *p<0.05, **p<0.01,
